# Redo-urethroplasty for the management of recurrent urethral strictures in males: a systematic review

**DOI:** 10.1007/s00345-019-02709-7

**Published:** 2019-03-15

**Authors:** Sara Jasionowska, Oliver Brunckhorst, Rowland W. Rees, Asif Muneer, Kamran Ahmed

**Affiliations:** 1 MRC Centre for Transplantation, Guy’s Hospital, King’s College London, London, UK; 2 Department of Urology, King’s College Hospital, London, UK; 3 Urology Department, University Hospital Southampton, NHS Foundation Trust, Southampton, UK; 4 Department of Urology, NIHR Biomedical Research Centre, University College Hospital, University College London Hospitals NHS Foundation Trust, London, UK

**Keywords:** Urethral stricture, Redo-urethroplasty, Failed urethroplasty, Repeat urethroplasty

## Abstract

**Purpose:**

Redo-urethroplasty is a challenge for any genitourethral surgeon, with a number of techniques previously described. This systematic review aims to identify the surgical techniques described in the literature and evaluate the evidence for their effectiveness in managing recurrent urethral strictures.

**Materials and methods:**

A systematic review of the MEDLINE and EMBASE databases from 1945 to July 2018 was performed and the urethroplasty procedures were classified according to the site and surgical technique. Primary outcomes included success rates measured via re-stricture rates and the post-op maximum urinary flow rate. Secondary outcomes included complication rates and patient-reported quality of life.

**Results:**

A total of 39 identified studies met the inclusion criteria. Twenty-two studies described the use of excision and primary anastomotic urethroplasty with success rates showing wide variability (58–100%). Success rates reported according to the site of the stricture also varied: bulbar (58–100%) and posterior (69–100%) recurrent strictures. One-stage substitution urethroplasty was described in 25 studies with success rates of 18–100%, with the best outcomes reported for bulbar (58–100%) and hypospadias-related (78.6–82%) strictures. Two-stage substitution urethroplasty was described in 12 studies with the success rates of 20–100%, with the best evidence related to hypospadias-related and posterior urethral strictures. The buccal mucosa graft was the graft source with the best evidence for substitution urethroplasty (18–100%).

**Conclusions:**

Trends of effectiveness were identified for redo-urethroplasty modalities in different locations. However, the current levels of evidence are limited to small observational studies, highlighting the need for further larger prospective data to evaluate different techniques used for recurrent urethral strictures.

**Electronic supplementary material:**

The online version of this article (10.1007/s00345-019-02709-7) contains supplementary material, which is available to authorized users.

## Introduction

Open reconstructive urethroplasty offers a cost-effective treatment modality for urethral strictures with excellent early success rates of 79–95% [1]. However, long-term results demonstrate that 14–42% of patients require additional treatment for recurrent strictures after primary surgical reconstruction [2]. Despite this, there are few standardised guidelines for the treatment of urethral stricture recurrence following urethroplasty, with practice varying widely between urologists [3].

Redo-urethroplasty and direct vision internal urethrotomy (DVIU) are the established techniques. DVIU remains the most commonly utilised initial method, as it offers a minimally invasive approach with fewer technical challenges, and allows endoscopic assessment of the recurrent stricture. Whilst identified as a valuable therapeutic option for short (< 1 cm) or bulbar recurrent strictures, high failure rates of up to 100% and complications such as bleeding and infection limit its use to older men, those unwilling to undergo an open reconstruction or patients with multiple comorbidities [4, 5]. Additionally, repeated endoscopic treatment can result in a chronic urethral stricture, requiring redo DVIU and self-dilatation at regular intervals throughout a man’s lifetime [6]. On the contrary, reported success rates of redo-urethroplasty range between 67 and 92% [1].

There is considerable variation in the treatment of recurrent urethral strictures from different groups and organisations [7]. Recurrent strictures remain challenging for reconstructive surgeons, as they are often more complex, associated with extensive scarring and poor tissue vascularity. Data suggest that prior endoscopic treatment is an independent risk factor for failure after urethroplasty [8]. Additionally, numerous urethroplasty techniques have been described in the literature, but there is a paucity of guidelines with regard the optimal choice of procedure for recurrent strictures, based on the stricture aetiology and location. Therefore, this systematic review aims to:

1. Identify the current techniques described in the literature for redo-urethroplasty for recurrent urethral strictures.
2. Evaluate the current evidence base for the effectiveness of different redo-urethroplasty techniques via re-stricture and complication rates.
3. Discuss current follow-up methods following redo-urethroplasty.
4. Suggest treatment options based on the current evidence for recurrent urethral strictures at different locations and of different aetiologies.

## Materials and methods

This systematic review was conducted according to the Preferred Reporting Items for Systematic Reviews and Meta-Analyses (PRISMA) guidelines [9]. Additionally, this study was prospectively registered on the PROSPERO database (Registration Number CRD42018088874).

### Study eligibility criteria

Original research articles including randomised controlled trials, case series and cohort studies as well as conference abstracts with sufficient data that described techniques and outcomes of redo-urethroplasty were included in this study.

Abstracts with insufficient information, non-English articles, case reports, paediatric studies or studies utilising female subjects were excluded. Additionally, only patients with at least one failed urethroplasty previously and having undergone a redo-urethroplasty were eligible for analysis.

### Information sources and search

Studies were identified by searching MEDLINE and EMBASE databases from 1945-July 2018. No restrictions with regard to publication status were imposed. The last search was performed on the 22.07.2018. Search terms included a combination of “urethroplasty” and “redo” or “reoperative”, which identified the majority of the articles. These results were combined with “hypospadias”, “urethral”, “panurethral”, “treatment failure”, “urethra surgery” and technique” to supplement the identified studies. A reference review of identified articles was subsequently carried out. Ongoing clinical trials were searched for grey literature at http://www.clinicaltrials.gov with authors of potentially relevant studies contacted for preliminary or unpublished results for inclusion in review.

### Study selection

The search and study selection were performed independently by two reviewers (SJ and OB) with any discrepancies discussed. The advice of the third reviewer was sought if these could not be resolved. Studies that were identified using the search terms were assessed for further evaluation through abstract review once duplicates were removed. Subsequently, a full text review allowed exclusion of the irrelevant studies.

### Data collection and data items

Data were extracted from all studies onto a pre-defined extraction sheet including the following: author, publication year, surgical technique, number of patients, age of patient, location and length of the stricture, previous treatment, and aetiology of the stricture.

Primary outcome measures to assess the effectiveness specific for each surgical technique included success rate measured as re-stricture rate and post-op maximum urinary flow rate (*Q*_max_). Secondary measures included complication rates, post-operative patient-reported quality of life (assessed through questionnaires and patient-reported symptoms) and follow-up method.

This process was performed by two researchers independently (SJ and OB) and any discrepancies were addressed. Quality Appraisal of Case Series Studies Checklist [10] was used to assess bias as the case series and case–control studies were the only type of papers identified in this review. This is a 20-item checklist developed and validated by the Institute of Health Economics to be used as a risk of bias assessment tool.

## Results

### Study selection

**Fig. 1:**
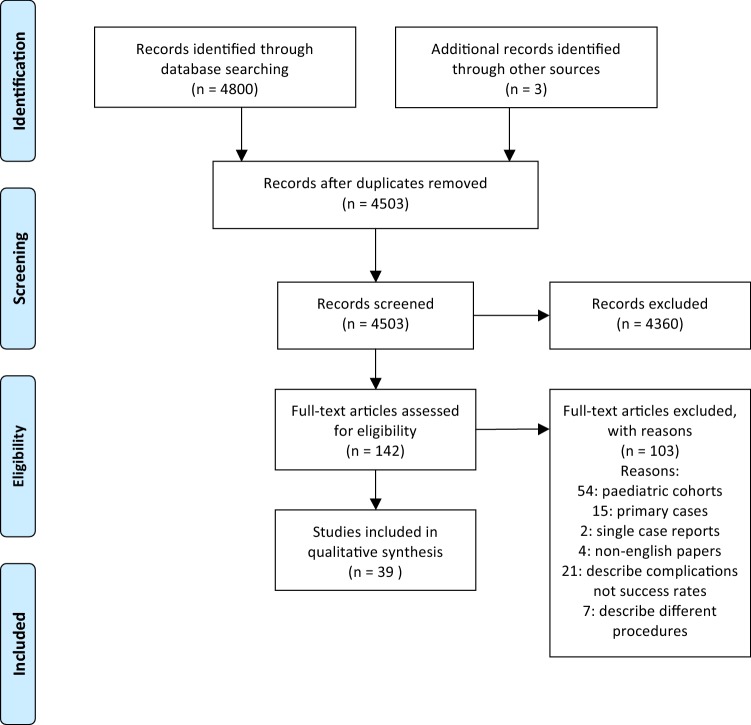
PRISMA diagram for study selection

A total of 4800 potentially relevant studies were identified. Abstract review and removal of duplications allowed for exclusion of 4661 articles. After review of full-texts, 103 studies were excluded from the analysis. Three articles were added after reference review. The final analysis was conducted on 39 articles (Fig. 1). Two ongoing clinical trials were identified with no data available for inclusion in review after authors were contacted.

### Study characteristics and results synthesis

Articles included in the review consisted of case series with both retrospective and prospective data analyses. The results were tabulated and the studies were grouped according to the surgical technique used and stricture location. Separate consideration was also given to strictures with a background of hypospadias and trauma. Data extracted from the studies were categorised into the following headings: the success rates of different redo-urethroplasty techniques, the success rates of different sites of stricture, complication rates and follow-up methods following redo-urethroplasty.

### Types of procedures and their outcomes

#### Anastomotic urethroplasty (AU)

Twenty-two studies described the use of anastomotic end-to-end urethroplasty in 893 patients (Table 1). AU was used to treat anterior strictures in 32% (*n* = 7) [11, 14, 17, 18, 20, 25, 29], posterior strictures in 45% (*n* = 10) [13, 15, 19, 23, 26, 28–30, 45, 46] and mixed bulbo-membranous strictures in 14% (*n* = 3) [15, 27, 29] of the studies which were reviewed. Five studies (*n* = 5) [12, 16, 21, 22, 24] did not provide information on the location of the stricture. Additionally, over half (*n* = 12) of the studies did not report the mean length of the urethral stricture treated, with only three studies [17, 27, 45] reporting the mean stricture length specifically for the patients who underwent the anastomotic procedure as 2.1 cm for bulbar, 2.8 cm for membranous and 4.2 cm for bulbo-membranous strictures. The mean follow-up ranged from 4 to 70 months in the studies reviewed. Six studies did not report the exact number of patients undergoing AU.

**Table 1:** Articles describing anastomotic urethroplasty

Article first author, publication date	No. of patients	Stricture site	Success rate	Failure definition	Mean follow-up (months)	Mean length (cm)
Ekerhult et al., 2016 [14]^a^	20	Bulbar	58% bulbar	Need for surgical instrumentation	Bulbar 70	Bulbar 2
Bhagat et al., 2011 [15]^a^	40	Bulbo-membranous/membranous/prostato-membranous	N/D	Maximum urinary flow < 15 ml/s	60	3.7
Gupta et al., 2008 [13]	52	Posterior	96.20%	Maximum urinary flow < 10 ml/s	54	N/D
Levine et al., 2014 [16]^a^	8	N/D	88%	Urethral patency < 16Fr	49	4.9
Blaschko et al., 2012 [12]^a^	54	N/D	88%	Need for surgical intervention or more than one endoscopic treatment	55	4.4
Siegel et al., 2015 [17]	19	Bulbar	95%	N/D	30.1	2.1
Barbagli et al., 1996 [11]^a^	2	Anterior	100%	N/D	57	N/D
Morey et al., 1997 [18]^a^	N/D	Bulbar	100%	N/D	12	N/D
Wadhwa et al., 1998 [19]^a^	14	Posterior	78.57%	Need for surgical instrumentation	4	2
Joseph et al., 2002 [20]^a^	N/D	Bulbar/penile	100%	N/D	60	N/D
Shau et al., 2015 [21]^a^	N/D	N/D	80%	Need for any instrumentation	42	2.7
Jakse et al., 1996 [22]	52	N/D	90.40%	N/D	45	N/D
Orabi et al., 2008 [23]^a^	25	Posterior	97%	N/D	N/D	N/D
Imbeault et al., 2014 [24]^a^	N/D	N/D	N/D	Radiological abnormality	25	N/D
Cavalcanti et al., 2012 [25]^a^	6	Bulbar	81.80%	Need for any instrumentation or *Q*_max_ < 15 ml/s	30.8	2.8
Pardeshi et al., 2016 [26]	21	Posterior	95.20%	Need for instrumentation	N/D	N/D
Kulkarni et al., 2015 [27]	15	Bulbo-membranous	93.30%	Need for any instrumentation	18	4.2
Patrascoiu et al., 2012 [28]	16	Posterior	68.70%	Need for instrumentation or maximum flow < 18 ml/s	38	N/D
Andrich et al., 2011 [29]^a^	N/D	Bulbar/membranous/prostatic	100% bulbar, 75% bulbo-prostatic	Radiologically abnormal	42	N/D
Webster et al., 1990 [30]^a^	20	Membranous	N/D	Need for any instrumentation	N/D	N/D
Shenfeld OZ, 2004 [45]	8	Membranous	100%	Need for any instrumentation	27	2.8
Kulkarni SB, 2018 [46]^a^	541	Posterior	79.13%	N/D	68	N/D

^a^Other techniques also described in the study

Success rates were reported in 19 studies and ranged from 58 to 100% (Table 1). The most commonly used definition for procedural failure was the need for any instrumentation as reported in 32% (*n* = 7) [21, 25–28, 30, 45] of studies. The need for surgical intervention reported in 14% (*n* = 3) [12, 14, 19] and radiological abnormality reported in 9% (*n* = 2) [24, 29] of studies represented the next most common definitions. 27% (*n* = 6) of studies did not report the failure definition adopted for the data analysis. However, the definition utilised showed differing success rates. When failure was defined as a need for any instrumentation, success rates ranged from 68.7 to 100%. The need for surgical intervention was associated with success rates of between 58 and 88%. Radiological recurrence rate was reported as 0–25%; however, this is a less useful measure, as re-intervention is also based on patients’ symptoms rather than radiological abnormality in isolation. 

#### One-stage substitution urethroplasty

Twenty-five studies (768 patients) described the use of one-stage substitution urethroplasty with a variety of grafts and flaps (Table 2). It was used to treat anterior urethral strictures (*n* = 18), posterior strictures (*n* = 5) and panurethral strictures (*n* = 1). Three studies reporting anterior strictures focused on hypospadias cases with 5 studies not discussing the location of the urethroplasty. The mean length of the stricture was reported in six studies with the mean length reported between 4 and 8 cm [32–34, 39, 41, 43].

**Table 2:** Articles describing one-stage substitution urethroplasty

Study	N. of patients	Stricture site	Graft type	Graft placement site	Success rate	Failure definition	Mean follow-up length (months)	Mean length (cm)
Ekerhult et al., [14]^a^	30	Penile Bulbar	BMG and fasciocutaneous	N/D	Bulbar 58% Penile 18%	Need for surgical intervention	Penile 82 Bulbar 70	Penile 4 Bulbar 2
Rosenbaum et al., [31]	50	Penile Bulbar	BMG	Bulbar ventral onlay, penile dorsal inlay	Bulbar 82% Penile 71.4 overall 80.8%	Need for any instrumentatio*n*/*Q*_max_ rate < 5 ml/s	25.6	N/D
Sevinc et al., [32]	24	Penile Bulbar	full thickness circumferential skin graft from arm (*n* = 20), inner thigh (*n* = 10) and abdomen (*n* = 4)	N/D	Arm graft 80%, inner thigh 60%, abdomen 50%, overall 69%	Need for surgical intervention	23.2	7.7
Javali et al., [35]^a^	21	Penile Bulbar	BMG	Dorsal onlay Ventral onlay	85.70%	Need for any instrumentation	42.4	3.18
Pfalzgraf et al., [36]^a^	33	Penile Bulbar	BMG and mesh	Bulbar—ventral onlay, penile—ventral onlay and dorsal onlay, dorsal inlay	Bulbar 88.2% Penile 68.8%	Need for any instrumentation	11.8	N/D
Levine et al., [16]^a^	26	Penile Bulbar Membranous	BMG and penile island flap	Onlay	Not discussed	Urethral patency < 16 Fr	49	4.9
Tang et al., [37]	4	Posterior	BMG	Inlay	50%	N/D	N/D	N/D
Blaschko et al., [12]^a^	130	N/D	BMG and faciocutaneous	Onlay tubularised	Not discussed	Need for surgical intervention or more than one endoscopic intervention	55	4.4
Barbagli et al., [11]^a^	12	Penile Bulbar	Free patch	N/D	100%	N/D	57	N/D
Morey et al., [18]^a^	N/D	Bulbar	BMG, penile skin	BMG unknown, penile circular fasciocutaneous flap	Patch graft 100%, penile circular rasciocutaneous flap = 79%	N/D	12	N/D
Wadhwa et al., [19]^a^	1	posterior	Forearm free flap	N/D	100%	Need for surgical intervention	4	2
Zargooshi et al., [38]	12	Hypospadias	Tunica vaginalis	Combined onlay tube	Not discussed	N/D	30	N/D
Xu et al., [39]	56	Hypospadias	Lingual, BMG	onlay	78.60%	Non-functional urethra	38.1	5.6
Barbagli et al., [40]^a^	21	Hypospadias	Penile skin/BMG	Penile flaps and dorsal inlay; BMG dorsal inlay/ventral onlay	Penile skin 80%, BMG 82%	Need for any instrumentation	33.8	N/D
Mehrsai et al., [41]	10	N/D	BMG	tubed graft	70%	Radiological abnormality	22	4.9
Joseph et al., [20]^a^	N/D	Penile Bulbar Panurethral	Scrotal skin, post-auricular skin, BMG	N/D	Barbagli patch 100% Orandi flap 75%	N/D	60	N/D
Mehrsai et al., [33]	34	Anterior Posterior	BMG	Tubed graft	76.50%	Need for any instrumentation	28	5.1
Shau et al., [21]^a^	N/D	Bulbar, penile	BMG and skin flaps	Penile—inlay, Bulbar—onlay	75% skin flaps, BMG unknown	Need for any instrumentation	42	2.7
Pandey et al., [34]	104	Anterior	BMG	Ventral onlay	91.40%	N/D	54	8
Imbeault et al., [24]^a^	N/D	N/D	BMG, scrotal skin, lingual graft	Dorsal/ventral	Not discussed	Radiological abnormality	25	N/D
Andrich et al., [25]^a^	23	Bulbar	BMG	Ventral onlay and dorsal onlay, double graft	81.80%	Need for any instrumentation or* Q*_max_ < 15 ml/s	30.8	2.8
Andrich et al., [42]^a^	40	N/D	N/D	N/D	N/D	N/D	96	N/D
Grant et al., [43]	39	anterior	BMG	Ventral onlay and dorsal onlay	87.20%	Urethral patency < 14 Fr	N/D	4
Andrich et al., [29]^a^	N/D	Bulbar Membranous	BMG, scrotal skin	BMG unknown, scrotal inlay	Bulbar BMG 100%, scrotal inlay 60%	Radiological abnormality	42	N/D
Vetterlein et al., [47]	98 (64 repeat, 34—2^°^	Bulbar, penile	BMG	Onlay, inlay	Repeat—87.5% 2°—70.8%	N/D	32	N/D

^a^Other techniques also described in the study

The total success rate was between 18 and 100%, at a mean follow-up between 4 and 82 months. Data on the success rate were missing from 5 of the studies. The most commonly utilised technique included the use of a buccal mucosal graft (BMG) in 80% of studies (*N* = 20). Three studies utilised a scrotal skin flap (scrotal inlay, Orandi flap, Barbagli patch) [20, 24, 29], with a further three using a penile skin (penile island flap) [16, 18, 40]. However, studies utilised a range of graft donor sites including abdominal skin [32], inner thigh skin [32], synthetic mesh [36], arm skin [32], post-auricular skin [20], lingual mucosa [24, 39], tunica vaginalis [38] and forearm free flap [19].

BMG grafts provided a success rate of 18–100% when used as a one-stage substitution in thirteen studies [11, 14, 25, 29, 31, 33, 34, 36, 37, 41, 43, 47]. The most commonly used definition for failure was the need for any instrumentation (28%, *n* = 7) with the success rate ranging from 68.8 to 88.2%. The presence of any radiological abnormality (12%, *n* = 3) provided a success rate of 60–100% with the need for surgical intervention (16%, *n* = 4) a success rate range of 18–100%. Eight studies did not state the definition of failure. The largest cohort investigating BMG urethroplasty was seen in a study by Pandey et al. [34] which described 104 cases of anterior urethral strictures with a mean length of 8 cm and treated with a BMG ventral onlay urethroplasty with good success rates of 91.4% at 54 months.

Different graft sources provided success rates of 80% for skin grafts from the arm, 60% inner thigh skin graft, 50% for abdominal skin graft as reported by one study by Sevinc et al. [32] and 60% for scrotal skin graft reported by Andrich et al. [29].

Moreover, Vetterlein et al. [47] described success rates of one-stage BMG urethroplasty for anterior strictures in secondary (re-operation using technique different to the one used in primary intervention) and repeat cases (re-operation using the same technique as used in primary intervention). Secondary procedures were successful in 87.5% of cases and secondary cases provided success rates of 70.8%. It was reported that a previous urethroplasty using any technique other than BMG urethroplasty had a significant negative impact on the outcome of the redo procedure.

#### Two-stage substitution urethroplasty

Twelve studies reported on 106 patients who had undergone a two-stage substitution urethroplasty procedure (Table 3). Five studies did not report the exact number of patients treated. It was used to treat anterior strictures in eight studies, posterior strictures in one and panurethral strictures in two studies. Three of the studies describing anterior strictures described only hypospadias cases and two did not report the exact site of the urethral stricture treated. Only one study described a mean stricture length of 8 cm (range 5–14 cm) [48].

**Table 3:** Articles describing two-stage substitution urethroplasty

Article first author, year of publication	N. of patients	Stricture site	Graft type	Graft placement	Success rate	Failure definition	Mean follow-up length (months)	Mean length (cm)
Ekerhult et al., 2016 [14]^a^	5	Penile	BMG	N/D	20%	need for surgical intervention	82	4
Javali et al., 2016 [35]^a^	N/D	Panurethral	lingual	Bracka technique	N/D	Need for any instrumentation	42.4	3.18
Pfalzgraf et al., 2014 [36]^a^	N/D	Bulbar Penile	BMG	N/D	N/D	Need for any instrumentation	11.8	N/D
Bhagat et al., 2011 [15]^a^	N/D	N/D	BMG	Scrotal inlay	N/D	Maximum urinary flow < 15 ml/s	60	3.7
Barbagli et al., 1996 [11]^a^	3	Bulbar penile	N/D	N/D	100%	N/D	57	N/D
Wadhwa et al., 1998 [19]^a^	3	Posterior	N/D	N/D	100%	Need for surgical intervention	4	2
Morrison et al., 2018 [44]^a^	27	Hypospadias	BMG Abdominal skin Post-auricular skin	N/D	100% for BMG	N/D	114	7
Barbagli et al., 2006 [40]^a^	N/D	Hypospadias	BMG/penile skin	N/D	50% for penile skin, 82% for BMG	Need for any instrumentation	33.8	N/D
Joseph et al., 2002 [20]^a^	N/D	Penile Bulbar Panurethral	BMG/post-auricular skin	N/D	78.9% for BMG, unknown for other	N/D	60	N/D
Calvacanti et al., 2012 [25]^a^	4	Bulbar Penile	BMG	N/D	N/D	Need for instrumentation of maximum urinary flow < 15 ml/s	30.8	2.8
Andrich et al., 2005 [42]^a^	49	N/D	N/D	N/D	N/D	N/D	96	N/D
Meeks et al., 2009 [48]	15	Hypospadias	BMG, abdominal skin, penile skin, posterior auricular skin	N/D	86%	Clinical evidence, increased post-void residual volume on USG	23	8

^a^Other techniques also described in the study

Eight studies utilised a BMG, with the remaining studies using post-auricular skin (*n* = 3), penile skin (*n* = 2), lingual mucosa (*n* = 1) and abdominal skin (*n* = 2) as a graft source. The total success rate ranged between 20 and 100% at a mean follow-up between 11.8 and 114-months in 12 studies [5, 11, 15, 19, 20, 25, 35, 36, 40, 42, 44, 48]. Five studies failed to report the success rates specific to this technique. Failure was defined as the need for any instrumentation in 4 (success rate range was 50–82%) and need for surgical intervention in 2 studies (success rate range of 20–100%). One study used clinical evidence and increased post-void residual urine volumes as failure definition; the success rate reported was 86%. Five studies did not define their success rates. Studies consisted of small cohorts with the biggest cohorts reported by Andrich et al. [42] and Morrison et al. with 49 and 27 cases, respectively [44].

### Stricture location, surgical procedures and their outcomes

Urethral strictures were classified into penile, bulbar, bulbo-prostatic, bulbo-membranous, hypospadias and posterior urethral strictures. Two techniques were described for penile strictures: one-stage and two-stage substitution urethroplasty (stricture length ranged from 1 to 12 cm). The success rates of 18–71.4%, at a mean follow-up of 25.6–82 months, were achieved for penile strictures treated with one-stage BMG urethroplasty in two studies [14, 31]. Poor success rate of 20% was achieved for two-stage BMG urethroplasty in one study [31]. Twenty-six articles were excluded from this part of the review due to a lack of site-specific success rate data.

Five studies described redo-urethroplasty as a treatment for recurrent bulbar strictures (stricture length range 1–8 cm). Techniques utilised included end-to-end anastomotic urethroplasty (four studies) and one-stage urethroplasty using a BMG (three studies) and fasciocutaneous penile flap (one study). Patients who received anastomotic urethroplasty achieved success in 58–100% of cases, at a mean follow-up of 12–82 months, as reported by four studies. The success rate of the substitution urethroplasty for bulbar strictures when using a BMG was 58–100%, at a mean follow-up of 25.6–82 months) as described by four studies. One study reported the success rate of 79% at a mean follow-up of 12 months for the penile circular fasciocutaneous flap (Table 4).

**Table 4:** The success rates of redo-urethroplasty for different urethral sites

First author and date published	Urethral location	Technique used	Success rate (%)	N. of patients	Mean follow-up (months)
Ekelhult et al., 2016 [14]	Penile	One-stage BMG/fasciocutaneous	18	n/a	82
Rosenbaum et., 2016 [31]	Penile	One-stage dorsal inlay BMG	71.40	n/a	25.6
		Two-stage BMG urethroplasty	20	n/a	25.6
Ekelhult et al., 2016 [14]	Bulbar	Anastomotic urethroplasty	58	n/a	70
		One-stage BMG	58	n/a	70
Siegel et al., 2015 [17]	Bulbar	Anastomotic urethroplasty	95	19	30.5
Rosenbaum et., 2016 [31]	Bulbar	One-stage ventral onlay BMG	82	n/a	25.6
Andrich et al., 2011 [29]	Bulbar	Anastomotic urethroplasty	100	n/a	42
		One-stage bulbar BMG	100	n/a	42
Morey et al., 1997 [18]	Bulbar	Anastomotic urethroplasty	100	n/a	12
		One-stage patch graft	100	n/a	12
		One-stage penile circular fasciocutaneous flap	79	n/a	12
Barbagli et al., 2006 [40]	Hypospadias	Anastomotic urethroplasty	87	n/a	33.8
		One-stage BMG urethroplasty	82	n/a	33.8
		One-stage penile skin flap	80	n/a	33.8
		Two-stage penile skin	50	n/a	33.8
		Two-stage BMG urethroplasty	82	n/a	33.8
Meeks et al., 2009 [48]	Hypospadias	Two-stage BMG urethroplasty	86	12	23
Morrison et al., 2018 [44]	Hypospadias	Two-stage BMG urethroplasty	100	n/a	114
Pandey et al., 2017 [34]	Anterior	One-stage BMG ventral onlay	91.40	104	54
Vetterlein et al., 2018 [47]	Anterior	One-stage BMG urethroplasty	Repeat 87.5 2°—70.8	Repeat 64 2°—34	32
Kulkarni et al., 2015 [27]	Bulbo-membranous	Anastomotic urethroplasty	93.30	15	18
Orabi et al., 2008 [23]	Posterior	Anastomotic urethroplasty	97	51	n/a
Pardeshi et al., 2016 [26]	Posterior	Anastomotic urethroplasty	95.20	21	n/a
Patrascoiu et al., 2012 [28]	Posterior	Anastomotic urethroplasty	68.70	16	38
Tang et al., 2011 [37]	Posterior	one-stage BMG inlay	50	4	n/a
Wadhwa et al., 1998 [19]	Posterior	Anastomotic urethroplasty	78.57	14	4
		Forearm free flap	100	1	4
		Two-stage urethroplasty	100	3	4
Gupta et al., 2008 [13]	Posterior	Anastomotic urethroplasty	96.20	52	54
Shenfeld et al., 2004 [45]	Posterior	Anastomotic urethroplasty	100	8	27
Kulkarni et al., 2018 [46]	Posterior	Anastomotic urethroplasty	79.13	541	68
Andrich et al., 2011 [29]	Bulbo-prostatic	Anastomotic urethroplasty	75	n/a	42
Webster et al., 1990 [30]	Bulbo-prostatic	Anastomotic urethroplasty	95	20	n/a

Only the end-to-end anastomotic urethroplasty technique was reported for the treatment of bulbo-prostatic and bulbo-membranous urethral strictures in two studies and one study, respectively. Stricture lengths varied between 1.5 and 7 cm for bulbo-prostatic strictures and 1–3 cm for bulbo-membranous strictures. Success rates ranged from 75 to 95% [29, 30] for bulbo-prostatic strictures and a 93.30% success rate was achieved for bulbo-membranous strictures in a single 15 patient case series [27].

Additionally, three studies described the success rates for different techniques used to treat the hypospadias-related strictures of the anterior urethra. The highest success rate was achieved for the two-stage BMG urethroplasty (success rates of 82–100% at mean follow-up of 23–114 months) as described by all three studies. Other urethroplasty techniques for hypospadias-related strictures were described by Barbagli et al. [40]; the anastomotic technique was successful in 87%, followed by one-stage BMG urethroplasty which was successful in 82%, one-stage penile skin urethroplasty with a success rate of 80% and two-stage penile skin urethroplasty with the success rate of only 50%, all at the mean follow-up of 33.8 months. However, as the technique is usually chosen depending on the severity of the stricture, the success rates may be influenced by selection bias which may reduce the importance of these differences in results reported.

Isolated posterior (membranous and prostatic urethra) strictures were treated utilising anastomotic, one-stage and two-stage redo urethroplasties. The anastomotic urethroplasty was reported as having successful outcomes in 68.7–100% of patients in seven studies. Only a single study with a small cohort of 4 cases was utilised for one-stage BMG urethroplasties which was successful in only 50% of cases (2/4 cases). Additionally, Wadhwa et al. [19] described the forearm flap graft as 100% successful in one patient and the two-stage substitution urethroplasty as 100% successful in three patients at 4 months follow-up.

### Complication rates and follow-up method post-redo-urethroplasty

Twenty-nine studies were included in this part of the analysis and the results are summarised in Table 5. Six studies used only one method for follow-up. These included isolated uroflowmetry in two, standardised questionnaires in one and urethrography in three studies. Most commonly, three methods of follow-up were combined, as reported by eight of the studies. Ten studies used a multitier follow-up approach using questionnaires (study-specific questionnaires or AUA symptom score) or uroflowmetry as an initial screen, with cystoscopy or urethrography only conducted if obstructive symptoms were seen. The cutoff point of maximum urinary flow triggering secondary investigations was set at 12 ml/s in two studies [27, 40], 14 ml/s in two studies [11, 33] and 15 ml/s in three studies [25, 34, 49]. Overall, the most commonly used method of follow-up, both in isolation and as part of multitest approach, was urethrography, as reported in 18 studies.

**Table 5:** Complication rates and follow-up method post-redo-urethroplasty

First author, data of publication	Mean follow (months)	Follow-up method	Mean time to recurrence (months)	Complication rate
Rosenbaum et al., 2016 [31]	25.6	Cystourethrography and cystoscopy annually	13.8	16.2% urge incontinence, 10.8% stress incontinence
Sevinc et al., 2016 [32]	23.2	Primary: uroflowmetry every 3 months Secondary: flexible cystoscopy and fluoroscopic imaging	N/D	Abscess, urethrocutaneous fistula
Javali et al., 2016 [35]	42.4	Primary uroflowmetry, post-void residual volume, urine culture and AUA symptom score every 4 months for the first 2 years then every 6 months, secondary: retrograde urethrography and urethroscopy	24.4	Wound infection in 9.52%, foot neuralgia in 4.76%, epididymo-orchitis in 4.76%, iatrogenic hypospadias meatus in 4.76%
Pfalzgraf et al., 2014 [36]	11.8	Standardised questionnaire	N/D	Urinary stress incontinence grade I in 15.2% and grade II in 6.1%, altered glans sensitivity in 24.2%, UTI in 36.1%
Bhagat et al., 2011 [15]	60	Uroflowmetry	N/D	Not discussed
Levine et al., 2014 [16]	49	Questionnaire and flexible cystoscopy	N/D	UTIi 10.2%, chordee 14.3%
Blaschko et al., 2012 [12]	55	Uroflowmetry, retrograde urethrography, voiding cystourethrography	17	Not discussed
Siegel et al., 2015 [17]	30.5	Uroflowmetry, voiding cystourethrography, questionnaire	N/D	Not discussed
Barbagli et al., 1996 [11]	57	Primary: uroflowmetry, urine culture, secondary: urethrography, voiding cystourethrography if uroflowmetry < 14 cc/s	N/D	Not discussed
Morey et al., 1997 [18]	12	Primary: questionnaires, secondary: retrograde urethrography	N/D	Not discussed
Wadhwa et al., 1998 [19]	4	Voiding cystourethrography	N/D	Not discussed
Zargooshi et al., 2004 [38]	30	Retrograde urethrography, cystourethroscopy, uroflowmetry, urethral biopsy	N/D	Not discussed
Morrison et al., 2018 [44]	114	Patient-reported symptoms, post-void residual volume	50.2	Not discussed
Xu et al., 2016 [39]	38.1	Uroflowmetry	N/D	Overall 21.4%: urethrocutaneous fistula and neourethral strictures
Barbagli et al., 2006 [40]	33.8	Primary: uroflowmetry, secondary: retrograde urethrography and urethroscopy if max flow < 12 cc/s	N/D	Not discussed
Mehrsai et al., 2005 [41]	22	Urethrography	3	Not discussed
Joseph et al., 2002 [20]	60	Retrograde urethrography, uroflowmetry, symptoms assessment	N/D	3% fistula, 3% wound infection, 12% post-void dribbling
Mehrsai et al., 2007 [33]	28	Primary: antegrade cystourethrography, questionnaires, urinalysis and culture, secondary: ultrasonography and cystourethrography if max flow < 14 cc/s	5.5	Fistula in 5.88%, erectile dysfunction in 2.94%, diverticulum in 2.94%, cheek swelling and perioral numbness in 17.6%, perioral wound infection 1.8%, UTI 23.5%
Shau et al., 2015 [21]	42	Primary: uroflowmetry, post-void residual volume, secondary: retrograde urethrography	N/D	Chronic leg pain 5%
Jakse et al., 1996 [22]	45	Patient-reported symptoms, clinical examination, urinalysis, uroflowmetry, voiding urethrography	N/D	Overall 9%: abscess, haematoma, wound infection, scrotal haematoma, epididymitis, temporary peroneal nerve palsy, incontinence
Pandey et al., 2017 [34]	54	Primary: questionnaire, post-void urine Volume, uroflowmetry, secondary: cystoscopy if max flow < 15 cc/s	N/D	Not discussed
Imbeault et al., 2014 [24]	25	Uroflowmetry, urethrography	3	Not discussed
Calvalcanti et al., 2012 [25]	30.8	Primary: uroflowmetry, secondary: urethrography, cystoscopy if max flow < 15 cc/s	N/D	Erectile dysfunction in 15.2%, ejaculatory symptoms in 21.2%
Kulkarni et al., 2015 [27]	18	Primary: uroflowmetry, urine culture, secondary: urethrography and urethroscopy if max flow < 12 cc/s	2	Not discussed
Patrascoiu et al., 2012 [28]	38	Clinical examination, post-void residual volume, uroflowmetry, urethrography	N/D	Epididymo-orchitis 18.75%, wound infection 12.5%, perinea haematoma in 12.5%, de novo erectile dysfunction in 6.25%
Andrich et al., 2011 [29]	42	Urethrography	N/D	Not discussed
Shenfeld et al., 2004 [45]	27	Urethrography (after 1 month), flexible retrograde urethroscopy (after year), uroflowmetry, post-void residual volume	N/D	UTI, bladder stones formation decreased erectile function in 12.5%, mild urinary incontinence in 12.5%
Myers et al., 2012 [49]	89	Primary: uroflowmetry, voiding cystourethrography; secondary: fluoroscopic imaging if urinary flow < 15 cc/s	N/D	Not discussed
Meeks et al., 2009 [48]	23	Clinical evidence, post-void residual volume	N/D	Minor voiding symptoms in 21%, fistula in 7%, mild dysuria in 21%, UTI 14%, BMG oral complications in 7%

Only four studies described details of the frequency of the follow-up regime. Uroflowmetry frequency varied at between 3 and 6 months in two studies [32, 35], with urethrography used one month post-operatively [45] and annually [31]. Average length of follow-up varied widely across studies between 4 and 114 months. The longest follow-up was 9.5 years reported by Morrison et al. [44]. Seven studies were followed up for less than 1 year, 14 for between 1 and 2 years, 6 for 2–3 years and 2 extended it to over 3 years. The most common complications described for each urethroplasty technique are summarised in Table 6.

**Table 6:** Procedure-specific complication rates post-redo-urethroplasty

Anastomotic urethroplasty	One-stage substitution urethroplasty	Two-stage substitution urethroplasty
De novo erectile dysfunction 12.5–18.75% [22, 28, 45]	UTI (36.1%) [36]	Voiding symptoms and mild dysuria (21%) [48]
Urinary incontinence (12.5%) [28, 45]	Altered glans sensitivity (24.2%) [36]	UTI (14%) [48]
Perineal haematoma (12.5%) [28, 45]	Cheek swelling and perioral numbness (17.6%) [33]	BMG oral complications (7%)

Eight studies described the mean time to recurrence of urethral stricture after redo surgery with ranges from two to 50.2 months [12, 24, 27, 31, 33, 35, 41, 44]. Six of these studies reported mean time to recurrence of less than 24 months [12, 24, 27, 31, 33, 41].

## Discussion

Recurrent urethral strictures are a surgical challenge due to the increased complexity of the strictures due to scarring and poor tissue vascularity. Whilst DVIU offers a less invasive initial treatment modality, it is associated with a high recurrence rate. This systematic review offers an overview of the current evidence for redo-urethroplasty in recurrent strictures, comparing the outcomes using different surgical techniques and according to varying stricture location.

The evidence for the use of anastomotic end-to-end urethroplasty has been assessed in 22 studies. Overall success rates vary widely across studies at 0–100%. When assessing its use to specific locations, anastomotic urethroplasty fared best when utilised in anterior bulbar and posterior recurrent strictures [11, 13, 14, 17–20, 23, 25, 27–29, 45, 46]. The success rates were reported as 58–100% and 68.7–100%, respectively, for these cohorts of patients, with four studies reporting success rates of 100% in anterior bulbar strictures.

The success rates for these stricture locations were reported as 75–95% [29, 30] and 93.30% [27], respectively.

The evidence for the use of one-stage substitution urethroplasty has been assessed in 25 studies. The overall success rate varied across the studies at 18–100%. The best results were obtained using the substitution urethroplasty technique for bulbar [14, 18, 25, 29, 31, 35, 36, 47] and hypospadias-related strictures [39, 40]. The success rates were reported at 58–100% and 78.6–82%, respectively, for these cohorts, with two studies reporting success rates of 100% in bulbar strictures. Conversely, the poorest outcomes were identified for posterior strictures with a success rate of only 50%; however, this consisted of a single four-patient study [37]. Studies assessing both penile and bulbar urethral strictures [14, 31, 36] achieved better results for the bulbar strictures with success rates of 18–71.40% and 58–88.2%, respectively. The BMG demonstrated the best evidence base as a graft source for more complex stricture recurrences when compared with penile fasciocutaneous flap [18] and scrotal skin [29], with 10 studies reporting success rates of over 80% [18, 25, 29, 31, 35–37, 40, 43, 47]. One-stage BMG urethroplasty was the most successful technique of all to treat penile strictures with a success rates of 71.40% [31].

Overall 12 studies assessed the use of two-stage substitution urethroplasty. The overall success rate varied across the studies at 20–100%, with the best results obtained in hypospadias-related and posterior strictures. The success rates were reported at 82–100% [40, 44, 48] and 100% [19], respectively, for these cohorts. However, the evidence for its use in posterior strictures is limited by a single small cohort study of three patients with a median stricture length of only 2 cm. On the contrary, this technique demonstrated the poorest outcomes in penile strictures with a success rate of 20% (mean stricture length 4 cm) [14]. However, results were quoted only for a small cohort consisting of five patients. The BMG was reported as a graft with the best evidence for two-stage substitution procedures and was superior to penile skin flap [40], with 3 studies reporting success rates of over 80% [40, 44, 48].

**Fig. 2:**
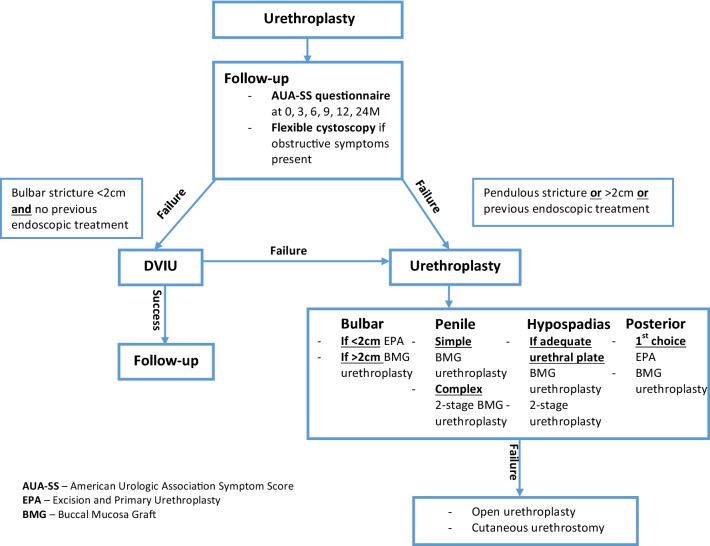
Summary of the most effective redo-urethroplasty techniques identified according to the stricture location

Based on the current best evidence and current guidelines identified, we have produced a suggested treatment algorithm for the management of recurrent urethral strictures (Fig. 2). Whilst the current evidence has demonstrated trends for treatment modalities according to the stricture location, it is important to consider that the identified evidence is limited in several factors.

All studies identified were level 4 evidence via case series or case–control studies only, with no randomised controlled studies identified. Furthermore, study cohorts were often retrospective and had limited follow-up with only 15 studies reporting over 40 participants. Formal risk of bias assessment conducted with use of the Quality Appraisal of Case Series Studies Checklist demonstrated that the majority of studies were prone to bias secondary to poor reporting on statistics and design. Most studies were single-centre retrospective case series with no formal statistical assessment, due to the small population size. Additionally, there was considerable study heterogeneity with broad aetiology inclusion criteria, with varying previous interventions and definitions of failures (Supplementary Table 1). Therefore, with this in mind it is important to consider the limitations of current trends in treatment and the widespread applicability of these results.

Finally, the choice of operation is based on the severity of the stricture. Single-stage procedures may be chosen for simple strictures, whereas more complex ones may be treated with two-stage surgery. This selection bias makes it virtually impossible to compare outcomes.

Understanding common complications for differing redo-urethroplasty techniques allows for patient-specific discussions pre-operatively (Table 6). The current literature demonstrates that common complications following end-to-end anastomotic urethroplasty include erectile dysfunction, urinary incontinence and perineal haematoma. When considering one-stage BMG urethroplasty, postoperative UTI, altered glans sensitivity, and complications of graft harvesting including cheek swelling and perioral numbness were the most common complications described. When using other graft sources, urethrocutaneous fistula and abscess formation were seen to be more common. Finally, two-stage BMG urethroplasty was most commonly complicated by voiding symptoms, dysuria, BMG oral complications and fistula formation.

Stricture recurrence was seen at less than 24 months in six out of eight studies in this review, with the longest time to recurrence of 50.2 months. Therefore, a standardised follow-up regime is required with an emphasis on this high-risk timeframe. Whilst the most cost-effective approach is difficult to establish from the current literature, previous evidence from a systematic review suggests a two-tier system to diagnose stricture recurrence [50]. A possible initial screening protocol could consist of the AUA-SS at every post-op visit, with flexible cystoscopy used as a second tier procedure if the obstructive symptoms are identified. Frequency of follow-up could be implemented on a 3-monthly basis initially with yearly follow-up after the first year (at 0 M, 3 M, 6 M, 9 M, 12 M and 24 M).

Current evidence is reliant on level 4 studies. Therefore, it is clear that further work is required. Whilst technically difficult to conduct due to the variability in surgical technique for individual strictures, more randomised controlled and standardised studies are required. There is a need for prospective data comparing the success rates of specific procedures at different stricture locations, utilising standardised definitions of success rates. This should additionally be compared to less invasive techniques such as DVIU. Strict inclusion criteria with regard to previous intervention and definitions of failure are needed. This will ensure that the future management decisions are patient-specific and based on the objective evidence rather than an institution preference.

We present the second systematic review on the outcomes of redo-urethroplasty, with a previous review of five studies, including paediatric cases conducted [51]. Success rates of between 35 and 85% out of a total of 212 redo-urethroplasty cases were identified. Through a broader search and inclusion criteria, we have been able to identify numerous further studies and, despite current paucity in high quality studies, are able to discuss trends in the evidence, with potential specific treatment modalities for strictures at different urethral sites. However, this review is not without its limitations. As mentioned previously, it is limited by the quality of the studies identified, with large heterogeneity and small cohort sizes meaning that results may not be applicable to all individual cases and no significant statistical pooling of results could be conducted. Additionally, this is a narrative systematic literature review leading to expert opinion. With all these limitations in mind, no definite conclusions may be drawn from the results. Even though the trends identified by this review are based on the best evidence available, we are uncertain whether suggested treatment modalities for different strictures guarantee the best possible outcomes.

## Conclusions

The success rates of redo surgery after failed urethroplasty are comparable to primary surgery when the appropriate technique is used. This review identified the possible optimal urethroplasty technique for different urethral stricture locations based on the limited current evidence in the literature. Anastomotic urethroplasty fared best when utilised in bulbar and posterior strictures. Substitution urethroplasty was most successful when BMG was used as a graft source. The best results for one-stage BMG urethroplasty were achieved when treating bulbar and hypospadias-related strictures. Multistage BMG urethroplasty was the most successful technique for hypospadias-related and posterior strictures. Redo-urethroplasty treatment and the follow-up pathway algorithm were designed based on the current evidence and existing guidelines. However, the use of Quality Appraisal of Case Series Studies Checklist revealed high risk of bias in the poor quality of studies identified in this review. Thus, we are uncertain whether the trends discussed are the most effective interventions for management of redo urethral strictures. This review highlights the limited current evidence with small cohorts demonstrating the need for further investigation in this difficult to manage group of patients.

## Electronic supplementary material

Below is the link to the electronic supplementary material.

Supplementary material 1 (DOCX 40 kb)
