# Supplementary material for: Redo-urethroplasty for the management of recurrent urethral strictures in males: a systematic review
Source: World J Urol. 2019 Mar 15;37(9):1801–15. doi: 10.1007/s00345-019-02709-7 (PMC6717180; doi:10.1007/s00345-019-02709-7)
Supplement: Supplementary file 1 — Supplementary material 1 (DOCX 40 kb) [file 345_2019_2709_MOESM1_ESM.docx]

**APPENDIX**

**Supplementary Table 1 - Risk of bias assessment tool applied to studies included in the review.**

| **Criterion** | | **Ekerhult et al, 2016** | **Shenfeld et al., 2004** | **Myers et al., 2012** | **Meeks et al., 2009** | **Rosenbaum et al, 2016** | **Sevinc et al., 2016**  **et al, 2016** | **Javali et al, 2016** | **Pfalzgraf et al, 2014** | **Bhagat et al, 2011** | **Gupta et al, 2008** | **Levine et al, 2014** | **Tang et al, 2011** | **Blaschko et al, 2012** | **Siegel et al, 2015** | **Barbagli et al, 1996** | **Morey et al, 1997** | **Wadhwa et al, 1998** | **Zargooshi et al, 2004** | **Morrison et al, 2018** | **Xu et al, 2016** |
| --- | --- | --- | --- | --- | --- | --- | --- | --- | --- | --- | --- | --- | --- | --- | --- | --- | --- | --- | --- | --- | --- |
| **Study objective** | **Was the hypothesis/aim/objective of the study clearly stated?** | **Y** | **Y** | **Y** | **Y** | **Y** | **Y** | **Y** | **Y** | **Y** | **Y** | **Y** | **Y** | **Y** | **Y** | **Y** | **Y** | **Y** | **P** | **Y** | **Y** |
| **Study design** | **Was the study conducted prospectively?** | **N** | **N** | **N** | **N** | **N** | **UC** | **N** | **N** | **UC** | **N** | **N** | **N** | **N** | **N** | **UC** | **N** | **N** | **N** | **N** | **N** |
|  | **Were the cases collected in more than one centre?** | **N** | **N** | **N** | **N** | **N** | **N** | **N** | **N** | **N** | **N** | **N** | **N** | **N** | **N** | **N** | **N** | **N** | **N** | **N** | **N** |
|  | **Were patients recruited consecutively?** | **Y** | **UC** | **Y** | **UC** | **Y** | **Y** | **Y** | **Y** | **Y** | **N** | **UC** | **UC** | **UC** | **UC** | **UC** | **N** | **N** | **N** | **N** | **Y** |
| **Study population** | **Were the characteristics of the patients included in the study described?** | **Y** | **P** | **Y** | **Y** | **Y** | **Y** | **Y** | **Y** | **Y** | **Y** | **Y** | **Y** | **Y** | **Y** | **Y** | **Y** | **Y** | **Y** | **Y** | **Y** |
|  | **Were the eligibility criteria (inclusion and exclusion criteria) for entry into the study clearly stated?** | **Y** | **Y** | **Y** | **Y** | **Y** | **Y** | **Y** | **Y** | **Y** | **Y** | **Y** | **Y** | **Y** | **Y** | **Y** | **Y** | **Y** | **Y** | **Y** | **Y** |
|  | **Did patients enter the study at similar point in the disease?** | **UC** | **Y** | **Y** | **Y** | **UC** | **Y** | **UC** | **Y** | **UC** | **UC** | **UC** | **UC** | **Y** | **UC** | **UC** | **UC** | **UC** | **UC** | **UC** | **UC** |
| **Intervention and cointervention** | **Was the intervention of interest clearly described?** | **Y** | **Y** | **Y** | **Y** | **Y** | **Y** | **Y** | **Y** | **Y** | **Y** | **Y** | **Y** | **Y** | **Y** | **Y** | **Y** | **Y** | **Y** | **Y** | **Y** |
|  | **Were additional interventions (cointerventions) clearly described?** | **Y** | **Y** | **Y** | **Y** | **Y** | **N** | **Y** | **N** | **Y** | **Y** | **UC** | **P** | **Y** | **UC** | **UC** | **Y** | **Y** | **Y** | **Y** | **Y** |
| **Outcome measures** | **Were relevant outcome measures established a priori?** | **Y** | **Y** | **Y** | **Y** | **Y** | **Y** | **Y** | **Y** | **Y** | **Y** | **Y** | **Y** | **Y** | **Y** | **Y** | **Y** | **Y** | **P** | **P** | **Y** |
|  | **Were outcome assessors blinded to the intervention that patients received?** | **N** | **N** | **N** | **N** | **N** | **N** | **N** | **N** | **N** | **N** | **N** | **N** | **N** | **N** | **N** | **N** | **N** | **N** | **N** | **N** |
|  | **Were the relevant outcomes measured using appropriate objective/subjective methods?** | **Y** | **Y** | **Y** | **Y** | **Y** | **Y** | **Y** | **Y** | **Y** | **Y** | **Y** | **Y** | **Y** | **Y** | **Y** | **Y** | **Y** | **Y** | **Y** | **Y** |
|  | **Were the relevant outcome measures made before and after the intervention?** | **Y** | **Y** | **Y** | **Y** | **Y** | **Y** | **Y** | **Y** | **Y** | **Y** | **Y** | **Y** | **Y** | **Y** | **Y** | **Y** | **Y** | **UC** | **Y** | **Y** |
| **Statistical analysis** | **Were the statistical tests used to assess the relevant outcomes appropriate?** | **Y** | **N** | **N** | **N** | **Y** | **Y** | **P** | **Y** | **Y** | **Y** | **Y** | **N** | **Y** | **Y** | **UC** | **UC** | **UC** | **UC** | **Y** | **UC** |
| **Study results and conclusions** | **Was follow-up long enough for important events and outcomes to occur?** | **Y** | **Y** | **Y** | **Y** | **Y** | **Y** | **Y** | **Y** | **Y** | **Y** | **Y** | **Y** | **Y** | **Y** | **Y** | **Y** | **Y** | **Y** | **Y** | **Y** |
|  | **Were losses to follow-up reported?** | **Y** | **Y** | **Y** | **Y** | **Y** | **Y** | **Y** | **N** | **N** | **N** | **N** | **Y** | **Y** | **UC** | **UC** | **N** | **N** | **Y** | **N** | **Y** |
|  | **Did the study provide estimates of random variability in the data analysis of relevant outcomes?** | **N** | **N** | **N** | **N** | **N** | **N** | **N** | **N** | **N** | **N** | **N** | **N** | **N** | **N** | **N** | **N** | **N** | **N** | **N** | **N** |
|  | **Were the adverse events reported?** | **Y** | **Y** | **Y** | **Y** | **Y** | **Y** | **Y** | **Y** | **Y** | **Y** | **Y** | **Y** | **Y** | **Y** | **Y** | **Y** | **Y** | **Y** | **Y** | **Y** |
|  | **Were the conclusions of the study supported by the results?** | **Y** | **Y** | **Y** | **Y** | **Y** | **Y** | **Y** | **Y** | **Y** | **Y** | **Y** | **Y** | **Y** | **Y** | **Y** | **Y** | **Y** | **Y** | **Y** | **Y** |
| **Competing interests and sources of support** | **Were both competing interests and sources of support for the study reported?** | **Y** | **N** | **N** | **N** | **P** | **P** | **Y** | **P** | **P** | **N** | **P** | **N** | **P** | **Y** | **N** | **N** | **N** | **P** | **P** | **Y** |
| **OXFORD CEBM – LEVEL OF EVIDENCE** | | **4** | **4** | **4** | **4** | **4** | **4** | **4** | **4** | **4** | **4** | **4** | **4** | **4** | **4** | **4** | **4** | **4** | **4** | **4** | **4** |

| **Criterion** |  | **Barbagli et al, 2006** | **Vetterlein et al., 2018** | **Kulkarni et al., 2018** | **Mehrsai et al, 2005** | **Joseph et al, 2002** | **Mehrsai et al, 2007** | **Shau et al, 2015** | **Jakse et al, 1996** | **Pandey et al, 2017** | **Orabi et al, 2008** | **Imbeault et al, 2014** | **Calvacanti et al, 2012** | **Andrich et al, 2005** | **Grant et al, 2017** | **Pardeshi et al, 2016** | **Kulkarni et al, 2015** | **Patrascoiu et al, 2012** | **Venkatesan et al, 2011** | **Webster at al, 1990** |
| --- | --- | --- | --- | --- | --- | --- | --- | --- | --- | --- | --- | --- | --- | --- | --- | --- | --- | --- | --- | --- |
| **Study objective** | **Was the hypothesis/aim/objective of the study clearly stated?** | **Y** | **Y** | **Y** | **Y** | **Y** | **Y** | **Y** | **Y** | **Y** | **Y** | **Y** | **Y** | **Y** | **Y** | **Y** | **Y** | **Y** | **Y** | **Y** |
| **Study design** | **Was the study conducted prospectively?** | **N** | **N** | **N** | **Y** | **UC** | **Y** | **N** | **UC** | **Y** | **N** | **N** | **N** | **N** | **N** | **UC** | **Y** | **N** | **N** | **N** |
|  | **Were the cases collected in more than one centre?** | **N** | **N** | **N** | **N** | **N** | **N** | **N** | **UC** | **N** | **N** | **N** | **N** | **N** | **N** | **N** | **N** | **N** | **N** | **N** |
|  | **Were patients recruited consecutively?** | **UC** | **Y** | **Y** | **UC** | **UC** | **UC** | **N** | **N** | **Y** | **N** | **N** | **N** | **N** | **UC** | **UC** | **UC** | **UC** | **UC** | **UC** |
| **Study population** | **Were the characteristics of the patients included in the study described?** | **Y** | **Y** | **Y** | **Y** | **Y** | **Y** | **Y** | **Y** | **P** | **Y** | **P** | **P** | **P** | **Y** | **Y** | **Y** | **Y** | **P** | **Y** |
|  | **Were the eligibility criteria (inclusion and exclusion criteria) for entry into the study clearly stated?** | **P** | **Y** | **N** | **P** | **Y** | **Y** | **Y** | **Y** | **P** | **P** | **Y** | **N** | **P** | **P** | **Y** | **Y** | **P** | **P** | **Y** |
|  | **Did patients enter the study at similar point in the disease?** | **UC** | **Y** | **Y** | **Y** | **Y** | **Y** | **UC** | **UC** | **Y** | **UC** | **UC** | **UC** | **UC** | **UC** | **UC** | **Y** | **UC** | **UC** | **UC** |
| **Intervention and cointervention** | **Was the intervention of interest clearly described?** | **Y** | **Y** | **Y** | **Y** | **Y** | **Y** | **Y** | **Y** | **Y** | **Y** | **Y** | **Y** | **Y** | **Y** | **Y** | **Y** | **Y** | **Y** | **Y** |
|  | **Were additional interventions (cointerventions) clearly described?** | **UC** | **Y** | **Y** | **Y** | **N** | **Y** | **N** | **Y** | **N** | **P** | **N** | **N** | **N** | **N** | **Y** | **Y** | **Y** | **N** | **Y** |
| **Outcome measures** | **Were relevant outcome measures established a priori?** | **Y** | **Y** | **Y** | **Y** | **Y** | **Y** | **Y** | **Y** | **P** | **UC** | **UC** | **Y** | **UC** | **Y** | **UC** | **Y** | **UC** | **UC** | **Y** |
|  | **Were outcome assessors blinded to the intervention that patients received?** | **N** | **N** | **N** | **N** | **N** | **N** | **N** | **N** | **N** | **N** | **N** | **N** | **N** | **N** | **N** | **N** | **N** | **N** | **N** |
|  | **Were the relevant outcomes measured using appropriate objective/subjective methods?** | **Y** | **Y** | **Y** | **Y** | **Y** | **Y** | **Y** | **Y** | **Y** | **Y** | **Y** | **Y** | **UC** | **Y** | **Y** | **Y** | **Y** | **UC** | **Y** |
|  | **Were the relevant outcome measures made before and after the intervention?** | **Y** | **Y** | **Y** | **Y** | **Y** | **Y** | **Y** | **Y** | **UC** | **Y** | **UC** | **UC** | **UC** | **UC** | **Y** | **Y** | **Y** | **UC** | **Y** |
| **Statistical analysis** | **Were the statistical tests used to assess the relevant outcomes appropriate?** | **N** | **Y** | **N** | **Y** | **UC** | **Y** | **Y** | **UC** | **UC** | **UC** | **UC** | **UC** | **UC** | **UC** | **UC** | **UC** | **UC** | **UC** | **Y** |
| **Study results and conclusions** | **Was follow-up long enough for important events and outcomes to occur?** | **Y** | **Y** | **Y** | **Y** | **Y** | **Y** | **Y** | **Y** | **Y** | **UC** | **Y** | **Y** | **UC** | **Y** | **Y** | **Y** | **Y** | **Y** | **Y** |
|  | **Were losses to follow-up reported?** | **N** | **N** | **N** | **N** | **Y** | **N** | **N** | **Y** | **Y** | **N** | **N** | **N** | **N** | **N** | **N** | **N** | **N** | **N** | **N** |
|  | **Did the study provide estimates of random variability in the data analysis of relevant outcomes?** | **N** | **N** | **N** | **N** | **N** | **N** | **N** | **N** | **UC** | **N** | **N** | **N** | **N** | **N** | **N** | **N** | **N** | **N** | **N** |
|  | **Were the adverse events reported?** | **Y** | **Y** | **Y** | **Y** | **Y** | **Y** | **Y** | **Y** | **Y** | **Y** | **Y** | **Y** | **Y** | **Y** | **Y** | **Y** | **Y** | **Y** | **Y** |
|  | **Were the conclusions of the study supported by the results?** | **Y** | **Y** | **Y** | **Y** | **P** | **Y** | **Y** | **Y** | **Y** | **P** | **P** | **Y** | **Y** | **N** | **P** | **Y** | **Y** | **Y** | **Y** |
| **Competing interests and sources of support** | **Were both competing interests and sources of support for the study reported?** | **N** | **N** | **P** | **P** | **N** | **N** | **Y** | **N** | **UC** | **P** | **N** | **P** | **N** | **N** | **N** | **P** | **N** | **N** | **N** |
| **OXFORD CEBM – LEVEL OF EVIDENCE** | | **4** | **4** | **4** | **4** | **4** | **4** | **4** | **4** | **4** | **4** | **4** | **4** | **4** | **4** | **4** | **4** | **4** | **4** | **4** |
